# 25-Hydroxyvitamin D_3_ supplementation improves growth performance and alleviates weaning stress in calves by modulating intestinal microbiota and metabolic pathways

**DOI:** 10.3389/fmicb.2026.1845847

**Published:** 2026-06-04

**Authors:** Wei Zhu, Xinyu Liu, Shuai Hu, Baoshan Feng, Yunfei Li, Xin Li, Fengtao Ma, Jianguo Li, Yanxia Gao, Hongjian Xu

**Affiliations:** 1 College of Animal Science and Technology, Hebei Agricultural University, Baoding, Hebei, China; 2 Key Laboratory of Healthy Breeding in Dairy Cattle (Co-Construction by Ministry and Province), Ministry of Agriculture and Rural Affairs, Baoding, Hebei, China; 3 College of Veterinary Medicine, Hebei Agricultural University, Baoding, Hebei, China

**Keywords:** 25-hydroxyvitamin D_3_, calf, metabolomics, microbial communities, weaning stress

## Abstract

The purpose of this study was to assess how 25-hydroxyvitamin D_3_ affected pre-and post weaning calves’ growth performance, weaning stress, fecal microbial communities and metabolites, and serum metabolites. A total of 30 healthy Holstein heifers were randomly assigned into a control group (6,100 IU VD_3_/ day; *n* = 15) and 25D group (2,100 IU VD_3_ + 4,000 IU 25(OH)D_3_/day; *n* = 15). The findings revealed that 25D considerably improved average daily gain (ADG) and starter intake during the entire experimental period and body weight on d56, 63, while body diagonal length and heart girth on d 63 were greatly increased (*p* < 0.05). In contrast to the control group, 25D reduced the levels of IL-1β, IL-6, TNF-*α*, and D-lactate acid (*p* < 0.05), and the higher levels of 25(OH)D_3_ and 1,25(OH)_2_D_3_ in serum on d 63 (*p* < 0.05). LEfSe analysis revealed that on day 56, 25D increased the relative abundance of butyrate producing bacteria (e.g., *g_Frisingicoccus* and *g_Lachnospiraceae_NK4A136*) and decreased the relative abundance of *g_Alistipes* associated with pro-inflammation (LDA > 2, *p* < 0.05). On d 63, 25D further increased the relative abundance of *g_Ruminococcus* and *g_Erysipelotrichaceae_UCG-003*. The metabolomic analysis showed that 25D significantly enriched key pathways in the fecal and serum, including alanine, aspartate and glutamate metabolism, cysteine and methionine metabolism, tryptophan metabolism, valine, arginine and proline metabolism, citrate cycle (TCA cycle), pyruvate metabolism, and fructose and mannose metabolism. The downregulation of fecal amino acid metabolites is consistent with the small intestine’s utilization of dietary amino acids, which may imply that 25D positively influences intestinal absorption and limits fecal amino acid accumulation. Concurrently, the upregulation of serum fumarate, (S)-malate, and citrate promotes energy metabolism. In conclusion, this study demonstrates that 25D improves calf growth and anti-inflammatory responses by modulating hindgut microbial communities, along with their energy and amino acid metabolic pathways. These findings elucidate the microbial and metabolic mechanisms through which 25D enhances calf health and productivity both before and after weaning, and alleviates weaning stress.

## 1 Introduction

The rearing of calves is important for the development of the cattle industry and is closely related to the future productive potential of cattle herds. The digestive system and immune function of suckling calves are not yet fully mature, which potentially constrains the establishment of intestinal microbiota, nutrient absorption, and immune regulation (Arrieta et al., 2014; Hulbert and Moisá, 2016). Moreover, weaning is a crucial phase in the growth of calves, during which the nutritional transition can lead to stress responses and intestinal dysfunction. Meanwhile, this period is also prone to causing oxidative stress, suppressing immune function, and triggering a systemic inflammatory response (Carroll et al., 2009), which disrupts the microbial balance and subsequently increases intestinal disease (Du et al., 2023). Intestinal health problems potentially resulted in growth retardation, increased mortality, and elevated economic losses in young animals (Moeser et al., 2017). Thus, seeking effective nutritional strategies to alleviate stress during suckling and weaning and improve calf health and intestinal problems is of great significance for achieving sustainable development in the cattle industry.

25-hydroxyvitamin D_3_ [25(OH)D_3_] is the primary metabolite of vitamin D_3_ (VD_3_) in the liver and has stronger biological activity than VD_3_ (Soares Jr et al., 1995). It possesses the ability to maintain the balance of calcium (Ca) and phosphorus (P), along with antioxidant, anti-inflammatory, and anticancer properties (Prietl et al., 2013; Tagliaferri et al., 2019). *In vivo*, 25-hydroxyvitamin D 1α-hydroxylase (CYP27B1) transforms 25(OH)D_3_ into the active form 1,25-dihydroxyvitamin D₃[1,25(OH)_2_D_3_]. Through association with the vitamin D receptors, it acts on various physiological processes such as bone development, the development of reproductive organs, inflammatory immune responses, and intestinal health, thereby mediating the pleiotropic effects of 25(OH)D_3_ (Merriman et al., 2018; Zhou et al., 2022; Cheng et al., 2023; Xiao et al., 2023). Previous research has demonstrated that acute bovine viral diarrhea virus infection can promote a massive release of pro-inflammatory factors and a sharp increase in acute phase proteins, leading to a significant decrease in serum 25(OH)D_3_ concentration, thereby revealing that inflammation can significantly affect vitamin D homeostasis in the body (Nonnecke et al., 2014). In addition, research reports that dietary supplementation with 25(OH)D_3_ not only improves mineral metabolism but also significantly alleviates LPS and weaning stress-induced oxidative damage and inflammatory responses in calves (Xu et al., 2021; Blakely et al., 2023). Additional research has revealed that 25(OH)D_3_ is also essential for intestinal homeostasis. In broiler chickens, supplementation of diets with 25(OH)D_3_ can maintain intestinal health by promoting intestinal development, optimizing the structure of microbial communities, and regulating immune function (Chou et al., 2009; Liang et al., 2019; Zhang et al., 2022). Therefore, supplementing with 25(OH)D_3_ as an effective nutritional regulator of certain critical physiological stages for calves can significantly improve their health status and enhance productivity.

Though 25(OH)D₃ demonstrates great potential in regulating intestinal homeostasis, immunity, and antioxidant function, the synergistic mechanisms of 25(OH)D₃ on the intestinal microbiota and metabolism, host metabolism, and systemic physiological functions have not been fully elucidated. We hypothesize that dietary supplementation with 25-hydroxyvitamin D₃ can promote growth performance and mineral absorption efficiency, alleviate weaning stress, optimize the structure and metabolism function of the gut microbiota, and improve blood metabolism in suckling calves, thereby comprehensively promoting their health. Therefore, the purpose of this investigation was aimed at the effects of supplementing with 25(OH)D₃ on calf growth performance, blood parameters, intestinal microbiota composition and metabolism, and serum metabolism. The findings may provide a theoretical basis for alleviating weaning stress, improving intestinal health, and enhancing growth performance in calves.

## 2 Materials and methods

All experimental procedures and animal manipulations were performed in accordance with the guidelines of the Institutional Animal Care and Use Committee of Hebei Agricultural University and approved (approval no.2024063).

### 2.1 Experimental design animals and management

The animal experiment was conducted between March 2025 and June 2025 at a commercial dairy farm (Fu Yuan Dairy Farm). The experiment was conducted as a randomized complete block design. Thirty healthy newborn Holstein heifers, with a mean aged of 1.62 days (SD = 0.49) and a mean body weight of 41.55 kg (SD = 2.89), were selected for this study. Calves were centrally raised until reaching 5 days of age to minimize differences. Thirty calves were randomly separated into two groups according to age and body weight (*n* = 15 per group): (1) milk replacer with 6,100 IU VD_3_ (control), (2) milk replacer with 2,100 VD_3_ + 4,000 IU 25(OH)D_3_ (25D group). This experiment customizes specialized milk replacer and calf starter to eliminate interference from other VD sources. Based on NASEM recommendations, all calves should consume 2,100 IU of conventional vitamin D₃ daily through milk replacer to meet their minimum vitamin D requirements and prevent deficiency (National Academies of Sciences Engineering and Medicine (NASEM), 2021). There were 500 KIU/g of vitamin D_3_ in the product (Zhongmu Biological Pharmaceutical Co., Ltd., Zhengzhou, China), and the 25D contained 250,000 IU/kg 25(OH)D_3_ (DSM Nutritional Products Ltd., Shanghai, China). This study lasted 63 days and was composed of 2 phases, comprising a 56-day pre-weaning period (1 to 56 days of age) followed by a 7-day post-weaning period (57 to 63 days of age). Within 1 hour of birth, calves were taken from their dams and placed in separate enclosures (1.6 × 2.4 m) with straw bedding. Within 2 hours of birth, all calves were given 4 L of colostrum (IgG ≥ 50 g/L) and another 2 L within 12 h. According to the farm feeding standards, calves were fed with milk replacer 2 times per day at 0500 and 1700 h with 6 L/d from d 1 to 7, 8 L/d from d 8 to 14, and 10 L/d from d 15 to 50. Weaning was carried out by reducing milk volume from day 50. Calves were fed 8 L/d from d 50 to 53 and 4 L/d from d 54 to 56. The milk replacer was mixed in 45 °C water and was cooled to 38 ± 1 °C for feeding. From day 3 of the experiment, calves had ad libitum access to oat grass and starter feed. All calves were housed in outdoor separate enclosures (1.6 × 2.4 m) with straw bedding. The average ambient temperature ranged from approximately 15 °C to 22 °C, and the average relative humidity ranged from approximately 55 to 65%. Calves had free access to water and calf starter (provided by Shijiazhuang Gushi Hongfa Biotechnology Co., Ltd., Shijiazhuang, China). Every day, all of the facilities and feeding equipment were cleaned. Table 1 describes the calf starter’s composition and nutrient levels.

**Table 1:** Ingredients and nutrient composition (%) of the calf starters.

Composition	
Ingredient (%)	
Corn	49.00
Soybean meal	25.00
Cottonseed meal	5.00
Wheat bran	7.10
DDGS	8.00
NaCl	0.60
Limestone powder	1.90
CaHPO4	0.50
Premix^1^	2.90
Total	100.00
Nutrition level	
Dry matter, %	90.23
Organic matter, %	91.46
Crude protein, %	20.73
Ether extract, %	3.50
Neutral detergent fiber, %	15.42
Acid detergent fiber, %	6.95
Ca, %	0.91
P, %	0.53

^1^The premix provided per kg of the starter was as follows: VA 12,000 to 14,500 IU, VE >250 IU, Cu 20 to 35 mg, Fe 100 to 150 mg, Mn 40 to 60 mg, Zn 60 to 80 mg, Se >0.7 mg, Co >0.9 mg.

### 2.2 Sample collection

#### 2.2.1 Growth measurements and feed intake

On days 56 and 63, measurements were taken of the body weight (BW) and body structures, including withers height, body length, heart girth, and body diagonal length. Average daily gain (ADG) was calculated as the change in BW between time points divided by the number of days. Based on the quantity offered and refused, the starting intakes were recorded daily. Starter samples were collected three times per week, mixed, and preserved at −20 °C for subsequent nutritional analysis. Feed efficiency was determined by dividing ADG by DMI (starter plus milk replacer intake).

#### 2.2.2 Blood and feces collection

On days 56 and 63, blood samples were taken for examination before to morning feeding. Using evacuated tubes devoid of anticoagulant, blood was drawn via jugular venipuncture. To extract the serum, the blood samples were centrifuged at 3,500 × g for 15 min at 4 °C. After that, the serum samples were then stored at −80 °C for further metabolomics and blood parameter study. At days 56 and 63, fecal samples were collected from 6 calves per group three times daily (at 0600, 1100, and 1800 h) for three consecutive days. The 6 calves were completely randomly selected from the 15 calves in each group, and it was ensured that they showed no statistically significant differences in initial body weight, age, and health status compared with the original group of 15 calves, thereby representing the overall metabolic characteristics of the group. On the final day of each sampling occasion, the samples from each individual calf were combined to form a composite fecal sample, which was then stored at −80 °C for subsequent analysis.

### 2.3 Analytical methods

#### 2.3.1 Serum parameters analysis

Standard commercial kits (Beijing Huaying Biotechnology Research Institute, Beijing, China) was used to measure serum biochemical parameters and concentrations of mineral elements: the serum glucose (GLU), total protein (TP), albumin (ALB), globulin (GLB), blood urea nitrogen (BUN), total cholesterol (TC), total antioxidant capacity (T-AOC), malondialdehyde (MDA), superoxide dismutase (SOD), catalase (CAT), diamine oxidase (DAO), IL-1β, IL-6, TNF-*α*, D-lactate, calcium (Ca), phosphorus(P), magnesium (Mg), vitamin D_3_ (VD_3_), 25-hydroxyvitaminD_3_ [25(OH)D_3_] and 1,25-dihydroxyvitamin D₃[1,25(OH)_2_D_3_].

#### 2.3.2 Feces nutrient analysis

Feeds and feces were analyzed according to the Association of Official Analytical Chemists (AOAC International, 2000). Samples were pulverized in a mill to fit through a 1-mm screen after being dried in a convection oven at 55 °C for 48 h. The contents of dry matter (DM), crude protein (CP), ash, and ether extract (EE) were analyzed in accordance with the methods of the Association of Official Analytical Chemists (AOAC International, 2000). According to Van Soest, neutral detergent fiber (NDF) and acid detergent fiber (ADF) were analyzed (Van Soest et al., 1991).

#### 2.3.3 DNA extraction, 16S rRNA gene sequencing, and bioinformatic analysis

Microbial genomic DNA was extracted from feces samples using the E. Z. N. A.® Soil DNA Kits (Omega Bio-Tek, Norcross, GA, US), according to the manufacturer’s instructions. The quantity and quality of the extracted DNA were measured using a NanoDrop2000 spectrophotometer (Thermo Fisher Scientific, United States) and 1% agarose gel electrophoresis, respectively. We amplified the V3 to V4 hypervariable regions of the 16S rRNA genes using the primer pairs 338F (5’-ACTCCTACGGGAGGCAGCAG-3′) and 806R (5’-GGACTACHVGGGTWTCTAAT-3′) on a T100 Thermal Cycler (BIO-RAD, United States), as described by Liu et al. (2016). Following demultiplexing, the sequences obtained were first subjected to quality filtering using fastp (0.19.6) (Chen et al., 2018) and subsequently merged using FLASH (v1.2.11) (Magoč and Salzberg, 2011). The DADA2 (Callahan et al., 2016) plugin in the QIIME2 (version 2024) (Bolyen et al., 2019) pipeline was then used to denoised the high-quality sequences using the suggested parameters. This method yields single-nucleotide resolution based on error patterns within samples. Typically, DADA2 denoised sequences are referred to as amplicon sequence variants (ASVs). The Naive Bayes consensus taxonomy classifier, integrated within QIIME2 and utilizing the SILVA 16S rRNA database (v138.2), was employed for the Taxonomic assignment of ASVs. Mothur v1.30.1 was used to compute rarefaction curves alpha diversity indices, including observed ASVs, Chao1, and Shannon index, based on the ASVs’ data (Schloss et al., 2009). By using the Vegan v2.5–3 package, principal coordinate analysis (PCoA) based on Bray–Curtis dissimilarity was conducted to assess the similarity among microbial communities across different samples. Using the Vegan v2.5–3 package, the PERMANOVA test was conducted to determine the percentage of variance explained by the therapy and its statistical significance. The linear discriminanCPt analysis (LDA) effect size (LEfSe)^1^ (Segata et al., 2011) was performed to identify the significantly abundant taxa (phylum to genera) of bacteria among the different groups (LDA score > 2, *p* < 0.05).

#### 2.3.4 Serum and feces metabolite profiling analysis

To extract metabolites, 200 μL of each serum sample was transferred into a 1.5 mL centrifuge tube, followed by the addition of 800 μL of a solution [acetonitrile: methanol = 1:1(v/v)] containing four internal standards (0.02 mg/mL L-2-chlorophenylalanine, etc.). The samples underwent vortex mixing for 30 s and were subsequently subjected to low-temperature sonication for 30 min at 5 °C with a frequency of 40 KHz. To precipitate the proteins, the samples were kept at −20 °C for 30 min. The samples were then centrifuged for 15 min at 4 °C and13000 g. After being extracted, the supernatant was blown dry using nitrogen. Subsequently, re-solubilized the sample in a 100 μL solution (acetonitrile: water = 1:1), it was extracted using low-temperature ultrasonication for 5 minutes at 5 °C and 40 KHz. Centrifugation was then performed for 10 minutes at 13000 g and 4 °C. A 2 mL centrifuge tube was filled with a 100 mg sample of feces and a 6 mm diameter grinding bead. Metabolite extraction was carried out using 800 μL of extraction solution [methanol: water = 4:1 (v/v)] containing four internal standards (0.02 mg/mL L-2-chlorophenylalanine, etc.).

After grinding the samples for 6 minutes at −10 °C and 50 Hz using the Wonbio-96c (Shanghai Wanbo Biotechnology Co., LTD) frozen tissue grinder, the samples were extraction using low-temperature ultrasound for 30 min at 5 °C and 40 kHz. The feces samples were centrifuged for 15 min at 4 °C and 13,000 g after being left at −20 °C for 30 min. The supernatant was transferred to sample vials for LC–MS/MS analysis.

As a part of the system conditioning and quality control process, a quality control sample (QC) was created through the pooling of equal volumes from all the samples. The QC samples underwent the same testing and disposed procedures as the analytic samples. Principal component analysis (PCA), orthogonal least partial squares discriminant analysis (OPLS-DA), and a seven-cycle interactive validation assessing the model’s stability were carried out using the R package “ropls” (Version 1.6.2). Based on the *p*-value produced by the Student’s *t*-test and the Variable importance in the projection (VIP) provided by the OPLS-DA model, the metabolites with VIP > 1, *p* < 0.05 were identified as significantly distinct metabolites. Through metabolic enrichment and pathway analysis utilizing the KEGG database^2^, differential metabolites between the two groups were mapped into respective biochemical pathways. These metabolites may be categorized based on their roles or the routes they participate in.

### 2.4 Statistical analysis

Before data analysis, normality was checked using SAS’s UNIVARIATE technique (version 9.4, SAS Institute Inc., Cary, NC). Initial data were included as covariates for the growth and blood metabolite analyses. The calf BW, ADG, feed intake, and blood biochemical indexes were statistically analyzed using the Mixed procedure of SAS (version 9.4, SAS Institute Inc., Cary, NC), including fixed effects of treatment and time, interaction effect of treatment and time, and random effect of calf. The results were presented as mean with standard error of the mean. The Wilcoxon rank-sum test was used to compare microbial phyla, genera, and species; a *p*-value ≤ 0.05 indicated statistical significance, and a trend of difference was identified as 0.05 ≤ *p* ≤ 0.10.

## 3 Results

### 3.1 Growth performance and structural growth

The results of growth performance are presented in Table 2. By days 56 and 63, calves fed 25(OH)D_3_ demonstrated a significant increase in body weight (*p* < 0.05). Meanwhile, from d 1 to 56, d 1 to 63, 25(OH)D_3_ increased starter intake (*p* < 0.05). In addition, 25(OH)D_3_ increased average daily gain from d 1 to 63 (*p* < 0.05), whereas there was no significant difference in feed efficiency (*p* > 0.05). As presented in Table 3, with respect to structural growth parameters, the body diagonal length and heart girth were significantly increased in the 25(OH)D_3_ at d 63 (*p* < 0.05).

**Table 2:** Effects of dietary 25(OH)D_3_ supplementation on feed intake and growth performance of calves.

Item	Control	25D	SEM	*p*-value
Body weight (kg)				
d 56	97.98	102.87	1.638	0.053
d 63	104.13	109.63	1.744	0.043
Starter intake (g/d)				
d 1 to 56	623.21	891.73	64.903	0.011
d 56 to 63	3343.73	3885.25	212.198	0.093
d 1 to 63	793.24	1078.82	71.921	0.014
ADG (kg/d)				
d 1 to 56	0.81	0.91	0.033	0.073
d 56 to 63	0.88	0.97	0.063	0.351
d 1 to 63	0.88	0.91	0.030	0.038
Feed efficiency, gain/DMI				
d 1 to 56	0.38	0.38	0.009	0.712
d 56 to 63	0.27	0.25	0.014	0.400
d 1 to 63	0.36	0.35	0.008	0.609

25D, 25-hydroxyvitamin D_3_; DMI, starter plus milk replacer intake; ADG, average daily gain; d, day; SEM, standard error of the mean.

**Table 3:** Effects of dietary 25(OH)D_3_ supplementation on structural growth of calves.

Item	Control	25D	SEM	*p*-value
Body height (cm)				
d 56	92.45	93.80	0.844	0.273
d 63	96.15	97.40	0.683	0.212
Body diagonal length (cm)				
d 56	85.90	84.30	0.858	0.204
d 63	90.80	92.95	0.674	0.037
Body length (cm)				
d 56	80.05	78.35	0.987	0.239
d 63	84.10	85.35	0.605	0.161
Heart girth (cm)				
d 56	96.65	98.60	1.067	0.213
d 63	102.35	105.40	0.908	0.029

25D, 25-hydroxyvitamin D_3_; d, day; SEM, standard error of the mean.

### 3.2 Serum biochemical indicators, antioxidant trait, cytokine contents, intestinal integrity, and vitamin D metabolite of calves

Table 4 showed the serum indexes between 25D and control on days 56 and 63. On day 63, 25D significantly decreased the MDA level (*p* < 0.05), but SOD activity was elevated (*p* < 0.05). The serum concentrations of the cytokines IL-1β, IL-6, and TNF-*α* were significantly lower in the 25(OH)D₃ group than in the CON group at 63 days (*p* < 0.05). Moreover, 25D significantly lowered D-lactate levels on days 56 and 63 (*p* < 0.05). As shown in Table 5, on days 56 and 63, the 25D significantly decreased the vitamin D_3_ levels compared with control (*p* < 0.01), while exhibiting significantly higher 25(OH)D₃ levels. Additionally, the concentration of 1,25(OH)₂D_3_ was significantly elevated in the 25(OH)D₃ group at d 63 (*p* < 0.05).

**Table 4:** Effects of dietary 25(OH)D_3_ supplementation on the serum biochemical indexes, antioxidant indexes, cytokine contents, and intestinal integrity of calves.

Item	Days	Control	25D	SEM	*p*-value
Biochemical indexes					
GLU (mmol/)	56	5.72	6.03	0.163	0.202
	63	5.77	6.01	0.156	0.302
TP(g/L)	56	56.78	56.17	0.712	0.557
	63	56.80	55.67	0.514	0.144
ALB(g/L)	56	34.61	34.36	0.567	0.757
	63	33.98	33.30	0.492	0.351
GLB(g/L)	56	22.16	21.81	0.671	0.716
	63	22.83	22.36	0.473	0.503
BUN (mg/dL)	56	10.99	12.20	0.704	0.248
	63	11.53	12.82	1.025	0.391
TC (mmol/L)	56	5.52	5.50	0.202	0.938
	63	5.54	5.55	0.183	0.987
Antioxidant indexes					
T-AOC (U/ml)	56	8.66	8.56	0.244	0.767
	63	7.80	8.08	0.479	0.694
MDA (mmol/L)	56	5.84	5.81	0.396	0.963
	63	6.52	5.43	0.341	0.043
SOD (U/ml)	56	164.51	171.70	8.813	0.574
	63	150.70	179.42	5.606	0.004
CAT (U/ml)	56	42.53	43.13	1.250	0.740
	63	43.24	43.67	2.056	0.883
Cytokine contents					
IL-1β (pg/ml)	56	26.49	24.87	1.314	0.401
	63	37.29	29.01	2.007	0.013
IL-6 (ng/ml)	56	63.79	62.72	2.882	0.796
	63	75.92	64.38	2.507	0.007
TNF-a (ng/ml)	56	313.68	299.25	18.510	0.592
	63	347.26	290.80	12.899	0.009
Intestinal integrity					
DAO (U/L)	56	2.70	2.53	0.173	0.495
	63	3.34	2.89	0.603	0.610
D-lactate(μg/L)	56	1320.52	1123.42	51.802	0.020
	63	1286.38	954.72	50.134	0.001

25D, 25-hydroxyvitamin D_3_; SEM, standard error of the mean; GLU, glucose; TP, total protein; ALB, albumin; GLB, globulin; BUN, urea nitrogen; TC, total cholesterol; T-AOC, total antioxidant capacity; MDA, malondialdehyde; SOD, superoxide dismutase; CAT, catalase; IL-1β, interleukin 1 beta; IL-6, interleukin 6; TNF-α, tumor necrosis factor alpha; DAO, diamine oxidase.

**Table 5:** Effects of dietary 25(OH)D_3_ supplementation on the vitamin D metabolite of calves.

Item	Days	Control	25D	SEM	*p*-value
Vitamin D metabolite					
Ca (mmol/L)	56	2.53	2.61	0.047	0.234
	63	2.54	2.67	0.045	0.064
P (mmol/L)	56	2.37	2.41	0.038	0.443
	63	2.41	2.48	0.071	0.553
Mg (mmol/L)	56	0.07	0.07	0.003	0.699
	63	0.06	0.07	0.004	0.665
VD_3_ (ng/mL)	56	5.33	1.82	0.202	<0.0001
	63	5.51	1.88	0.097	<0.0001
25D (ng/mL)	56	48.26	95.41	4.137	<0.0001
	63	54.63	97.30	4.792	<0.0001
1,25(OH)₂D_3_(pg/ml)	56	85.61	98.04	5.426	0.131
	63	87.09	102.83	3.444	0.007

25D, 25-hydroxyvitamin D_3_; SEM, standard error of the mean; Ca, Calcium; P, Phosphorus; Mg, Magnesium; VD_3_, Vitamin D_3_; 1,25(OH)₂D_3_, 1,25-Dihydroxyvitamin D_3_.

### 3.3 Effect of 25(OH)D₃ on feces bacterial communities in calves

**Figure 1:**
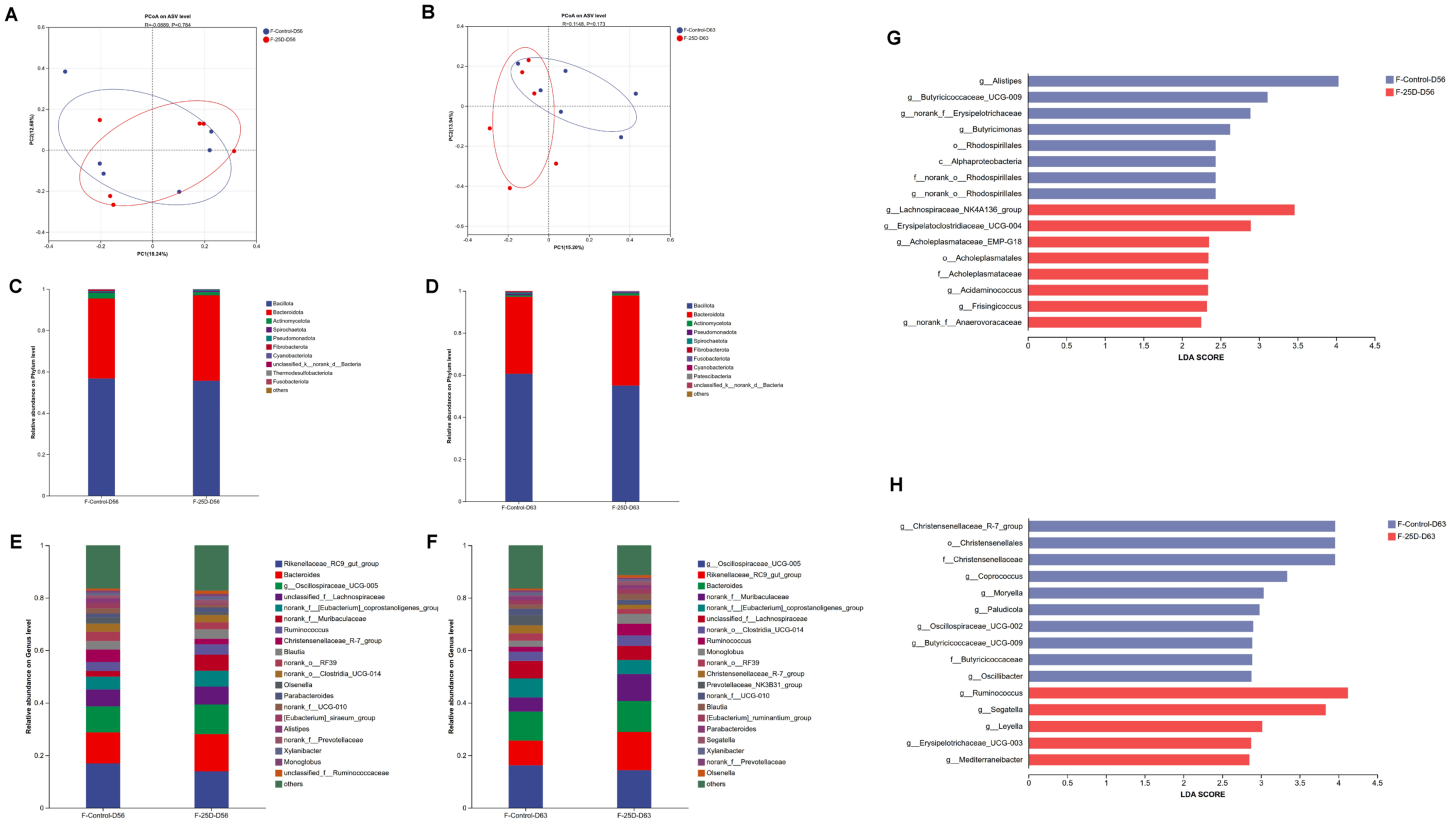
Effects of the 25(OH)D_3_ on feces microbiota of calves. **(A,B)** PCoA score plots of the bacterial compositional profiles for F-Control-D56 versus F-25D-D56 and F-Control-D63 versus F-25D-D63, comparisons, respectively. Distribution of feces bacterial taxa at the phylum **(C,D)** and genus **(E,F)** levels in calves. Different colors of bars represent different species, while the width of the bars represents the relative abundance of each phylum and genus. **(G,H)** Linear discriminant analysis effect size (LEfSe) analyses indicating differences in feces bacterial community composition between groups, with a linear discriminant analysis (LDA) score > 2 and a *p*-value < 0.05. CON, control group (*n* = 6); 25D, fed the 25-hydroxyvitamin D_3_ group (*n* = 6); F, feces; D, Day.

Table 6 presents the α-diversity indices of fecal bacteria at d56 and 63. On days 56 and 63, no significant differences were observed in the Chao1, Ace, Shannon, and Simpson indices of the fecal microbial community when comparing the Control and 25D groups (*p* > 0.05). Meanwhile, principal coordinates analysis (PCoA) based on Bray-Curtis distances revealed that the addition of dietary 25D had no effect on the overall structure of the fecal microbial composition on days 56 and 63 (Figures 1A,B).

**Table 6:** Effects of dietary 25(OH)D_3_ supplementation on the alpha diversity indices of calves.

Item	Control	25D	SEM	*p*-value
d 56				
Shannon	4.53	4.56	0.155	0.884
Simpson	0.04	0.04	0.011	0.898
Ace	403.28	411.75	29.358	0.842
Chao	403.18	412.11	29.334	0.834
d 63				
Shannon	4.84	4.45	0.147	0.091
Simpson	0.02	0.04	0.007	0.154
Ace	436.80	380.83	27.854	0.186
Chao	436.79	380.72	27.835	0.185

25D, 25-hydroxyvitamin D_3_; d, day; SEM, standard error of the mean.

As shown in Figure 1C–F, the bacterial species composition of fecal samples is displayed at the phylum (top 10) and genus (top 20) levels. At the phylum level, *Bacillota* and *Bacteroidota* were the dominant phyla in the intestinal microbiota, a pattern that was consistent between the Control and 25D groups on days 56 and 63 (Figures 1C,D). At the genus level, *g_Rikenellaceae_RC9_gut_group*, *g_Bacteroides* and *g_Oscillospiraceae_UCG-005* were the dominant genera across both experimental groups (CON and 25D) on days 56 and 63 (Figures 1E,F).

To better understand the dominance of specific bacteria in the CON and 25D groups, we used the linear discriminant analysis effect size (LEfSe) method for analysis (LDA > 2, *p* < 0.05). LEfSe analysis of feces bacterial communities on day 56 revealed that *g_Alistipes*, *o_Rhodospirillales*, *f_Butyricicoccaceae_UCG-009*, and *g_Butyricimonas* were significantly lower in the 25D group, while *g_Acidaminococcus*, *g_Lachnospiraceae_NK4A136_group*, *g_Frisingicoccus*, and *f_Erysipelatoclostridiaceae_UCG-004* were higher in the 25D group. On d 63, *g_Coprococcus*, and *g_Moryella* were significantly lower in the 25D group, while *g_Ruminococcus*, *g_Erysipelotrichaceae_UCG-003*, *g_Mediterraneibacter*, and *g_Leyella* were higher in the 25D group. (Figures 1G,H).

### 3.4 Feces microbiota metabolic

**Figure 2:**
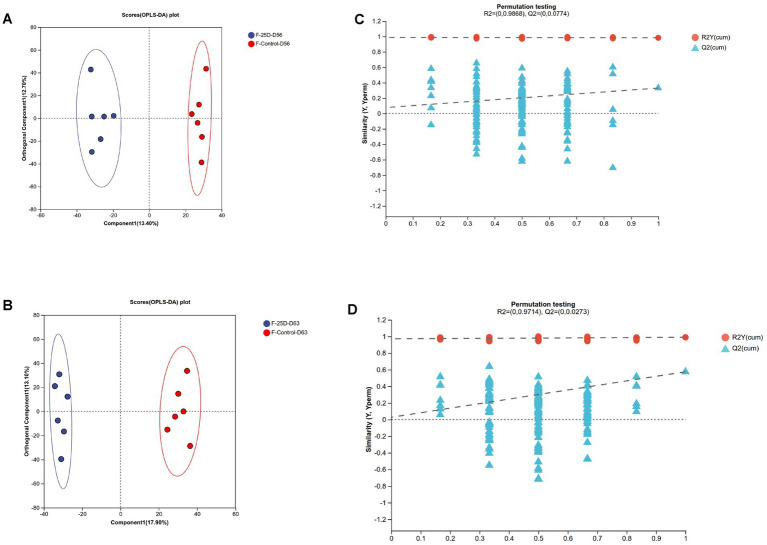
Effects of 25(OH)D_3_ on the feces metabolic profiles of calves. **(A,B)** OPLS-DA score plots in mixed ion mode for F-Control-D56 versus F-25D-D56 and F-Control-D63 versus F-25D-D63, F-Control-D56 versus F-25D-D56, F-Control-D63 versus F-25D-D63 comparisons, respectively. **(C,D)** Permutation test of the OPLS-DA model. Control, control group (*n* = 6); 25D, fed the 25-hydroxyvitamin D_3_ group (*n* = 6); F, feces; D, Day.

Figures 2A,B shows the OPLS-DA score plots of fecal metabolites from calves, illustrating the clustering patterns of the CON group and 25D group during the pre-and post-weaning periods. The findings revealed that, across different treatments, the samples exhibited a well-dispersed distribution, whereas within each specific treatment, they tended to cluster together, demonstrating that 25D supplementation distinctly shaped the fecal metabolic profiles. Additionally, all the blue Q2 points positioned from left to right were observed to be lower in value compared to the original blue Q2 point located at the far right (Figures 2C,D). Additionally, the regression line crossed below zero on the vertical coordinate axis less frequently. Both plots confirmed the reliability and validity of the results.

**Figure 3:**
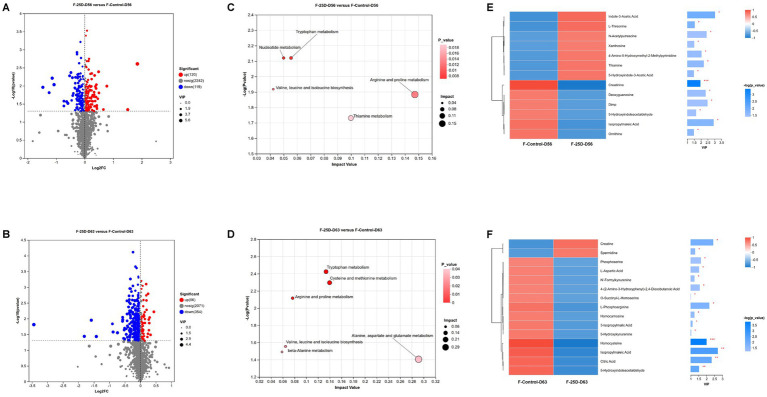
Identification of feces metabolites and metabolic pathways of calves. **(A,B)** Volcano plots of differential metabolites in mixed mode for the F-25D-D56 versus F-Control-D56 and F-25D-D63 versus F-Control-D63 comparisons, respectively. **(C,D)** Pathways of differential metabolites of the F-25D-D56 versus F-Control-D56 and F-25D-D63 versus F-Control-D63. Each bubble in graphs **(C,D)** represents a KEGG pathway (*p* < 0.05), and the larger the bubble, the more important the pathway. **(E,F)** The clustering heatmap represents the relative expression of differential metabolites (*p* < 0.05, VIP > 1) of the F-25D-D56 versus F-Control-D56 and F-25D-D63 versus F-Control-D63. Each row of the clustering heatmaps in graphs **(E,F)** represents a metabolite, and the color represents the relative expression amount of the metabolite in each group; the VIP bar chart represents the contribution value of the metabolite to the difference between the two groups; the bar color represents the significance of the metabolite in the two groups (* means *p* < 0.05, ** means *p* < 0.01, *** means *p* < 0.001). VIP, variable importance in projection; Control, control group (*n* = 6); 25D, fed the 25-hydroxyvitamin D_3_ group (*n* = 6); F, feces; D, Day.

Metabolites with *p* < 0.05 and VIP > 1 were considered significantly different. We visualized the differential metabolites among the groups via volcano plots. Analysis of 2,481 detected metabolites revealed distinct regulation patterns: before weaning, 120 were significantly upregulated and 119 downregulated; postweaning, this pattern shifted markedly to 56 upregulated and 354 downregulated (Figures 3A,B).

To further investigate the similarities and differences in the feces metabolites between the groups, clustering heatmap analysis was performed. During the pre-weaning period, 25D supplementation upregulated the fecal concentrations of N-acetylputrescine, thiamine, 5-hydroxyindole-3-acetic acid, and indole-3-acetic acid, while downregulating those of ornithine, creatinine, and 5-hydroxyindoleacetaldehyde. During the post-weaning period, 25D upregulated the feces concentrations of spermidine and creatine, downregulated the feces concentrations of L-aspartic acid, citric acid, homocysteine, phosphoserine, 5-hydroxyindoleacetaldehyde, and 5-hydroxykynurenine (Figures 3E,F).

KEGG topological analysis of differential metabolites among the groups indicated that 5 KEGG metabolic pathways were enrich in the pre-weaning group, primarily including arginine and proline metabolism, thiamine metabolism, tryptophan metabolism, nucleotide metabolism, and valine, leucine, and isoleucine biosynthesis (Figure 3C). Post-weaning enriched 6 KEGG metabolic pathways, primarily including alanine, aspartate and glutamate metabolism, cysteine and methionine metabolism, tryptophan metabolism, arginine and proline metabolism, valine, leucine and isoleucine biosynthesis nucleotide metabolism, and beta-Alanine metabolism (Figure 3D). Among these, 25D supplementation notably altered the levels of several tryptophan-related metabolites, including 5-hydroxyindole-3-acetic acid, indole-3-acetic acid, and 5-hydroxykynurenine, suggesting a potential modulatory effect on tryptophan metabolism pathways.

### 3.5 Serum metabolic

**Figure 4:**
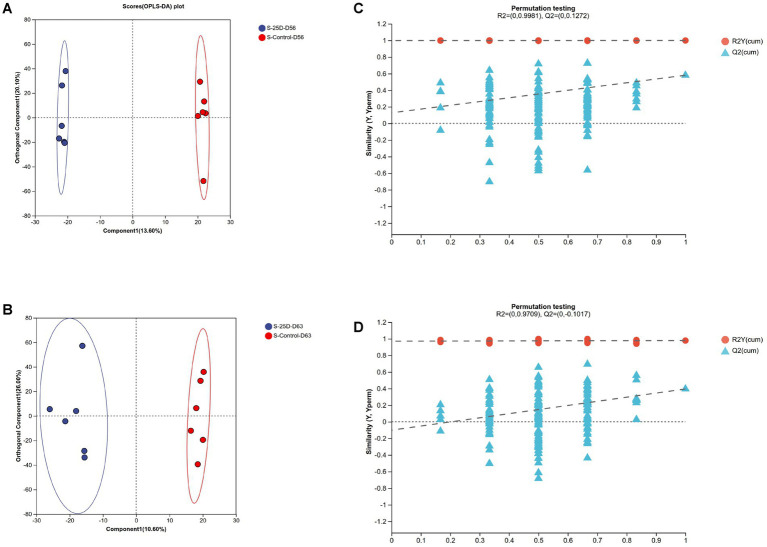
Effects of 25(OH)D_3_ on the serum metabolic profiles of calves. **(A,B)** OPLS-DA score plots in mixed ion mode for S-25D-D56 versus S-Control-D56 and S-25D-D63 versus S-Control-D63, S-25D-D56 versus S-Control-D56, S-25D-D63 versus S-Control-D63 comparisons, respectively. **(C,D)** Permutation test of the OPLS-DA model. Control, control group (*n* = 6); 25D, fed the 25-hydroxyvitamin D_3_ group (*n* = 6); S, serum; D, Day.

OPLS-DA was used to identify differential metabolites, and a permutation test was conducted to evaluate the stability of the OPLS-DA model. As shown in Figures 4A,B, the OPLS-DA score scatter plot showed significant differences between the CON group and 25D group during the pre-and postweaning periods, which indicated that the dietary supplementation with 25(OH)D_3_ altered the serum metabolites of calves. Additionally, the permutation test demonstrated the stability of the OPLS-DA model (Figures 4C,D), indicating that the OPLS-DA model was of good quality and not overfitted.

**Figure 5:**
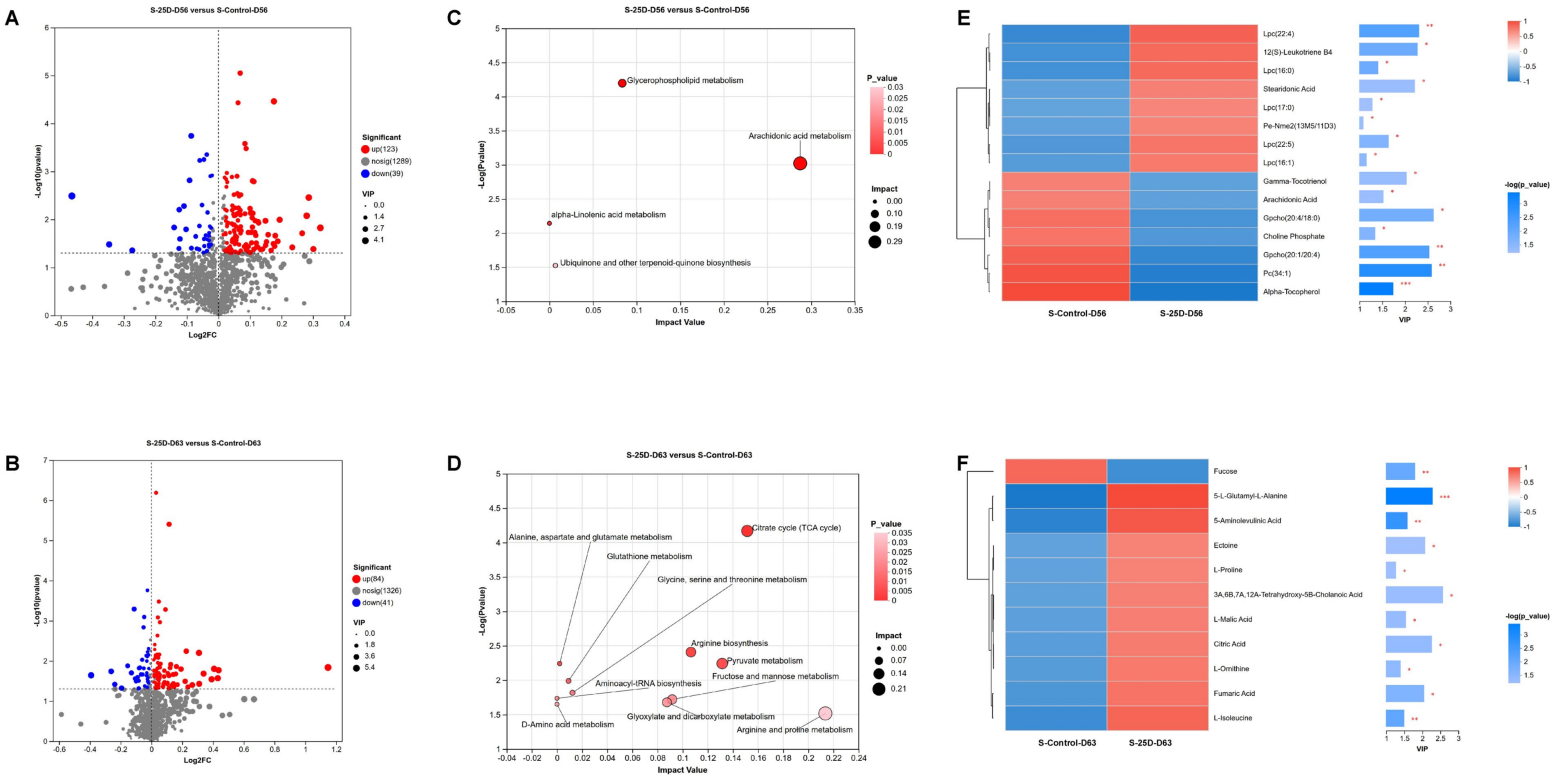
Identification of serum metabolites and metabolic pathways of calves. **(A,B)** Volcano plots of differential metabolites in mixed mode for the S-25D-D56 versus S-Control-D56 and S-25D-D63 versus S-Control-D63 comparisons, respectively. **(C,D)** Pathways of differential metabolites of the S-25D-D56 versus S-Control-D56 and S-25D-D63 versus S-Control-D63. Each bubble in graphs **(C,D)** represents a KEGG pathway (*p* < 0.05), and the larger the bubble, the more important the pathway. **(E,F)** The clustering heatmap represents the relative expression of differential metabolites (*p* < 0.05, VIP > 1) of the S-25D-D56 versus S-Control-D56 and S-25D-D63 versus S-Control-D63. Each row of the clustering heatmaps in graphs **(E,F)** represents a metabolite, and the color represents the relative expression amount of the metabolite in each group; the VIP bar chart represents the contribution value of the metabolite to the difference between the two groups; the bar color represents the significance of the metabolite in the two groups (*means *p* < 0.05, **means *p* < 0.01, ***means *p* < 0.001). VIP, variable importance in projection; Control, control group (*n* = 6); 25D, fed the 25-hydroxyvitamin D_3_ group (*n* = 6); S, serum; D, Day.

Based on the observed changes in serum metabolism, we performed an in-depth analysis of the serum metabolome. OPLS-DA analysis further revealed significant differences between the two groups. In total, the study detected 1,451 blood metabolites, with 123 significantly upregulated and 39 downregulated before weaning, while 84 were significantly upregulated and 41 downregulated after weaning (Figures 5A,B).

More specifically, the pre-weaning period upregulated the serum concentrations of 12(S)-leukotriene B4, Lpc (16:0), Lpc (17:0), Lpc (22:4), Lpc (22:5), Lpc (16:1), and stearidonic acid, while downregulated those of arachidonic acid, choline phosphate, alpha-tocopherol, and gamma-tocotrienol. In contrast, the post-weaning period upregulated the serum concentrations of L-ornithine, L-proline, citric acid, fumaric acid, L-malic acid, ectoine, 5-aminolevulinic acid, 5-L-glutamyl-L-alanine, and L-isoleucine, while downregulating that of fucose (Figures 5E,F).

The KEGG topological analysis of the differential metabolites among the groups indicated that a 25D supplement enriched 15 KEGG metabolic pathways. Specifically, the pre-weaning period primarily includes arachidonic acid metabolism, glycerophospholipid metabolism, ubiquinone and other terpenoid-quinone biosynthesis, and alpha-Linolenic acid metabolism. The post-weaning period enriched 11 KEGG metabolic pathways, namely arginine and proline metabolism, citrate cycle (TCA cycle), pyruvate metabolism, arginine biosynthesis, fructose and mannose metabolism, glyoxylate and dicarboxylate metabolism, glycine, serine and threonine metabolism, glutathione metabolism, alanine, aspartate and glutamate metabolism, aminoacyl-tRNA biosynthesis, and D-amino acid metabolism (Figures 5C,D). Collectively, these serum metabolite changes indicate that 25D supplementation exerts effects on multiple metabolic pathways, including lipid metabolism (e.g., arachidonic acid and glycerophospholipid metabolism) during the pre-weaning period, and TCA cycle during the post-weaning period.

### 3.6 Correlation between microbial genera and metabolites in feces

**Figure 6:**
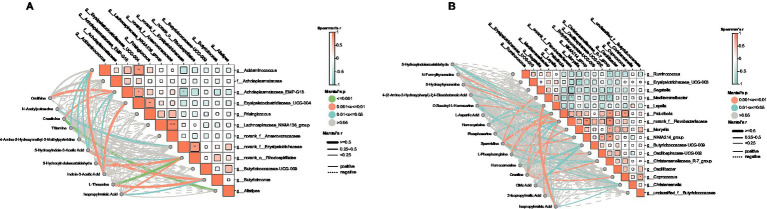
Correlation analysis of key microbiota with feces differential metabolites on days 56, 63 in the experiment. Mantel’s and Spearman’s correlation analyses of key microbiota with apparent differential metabolites. **(A)** Correlation analysis between key microbiota and differential metabolites in calf feces on day 56. **(B)** Correlation analysis between key microbiota and differential metabolites in calf feces on day 63. The thickness of the lines represents the correlation coefficient, with bold lines indicating Mantel’s *r* ≥ 0.5. The color of the lines represents significance, with green indicating highly significant correlation (*p* < 0.001), orange indicating significant correlation (0.001 < *p* < 0.01) and blue indicating significant correlation (*p* < 0.05). Spearman’s test was used for correlation analysis between key genera. The size and color gradient of the boxes represent Spearman correlation, with orange indicating positive correlation, blue indicating negative correlation (*means *p* < 0.05, **means *p* < 0.01, ***means *p* < 0.001).

To investigate the relationships between 25D induced changes in microbial communities and metabolic profiles, we performed correlation analysis between differential bacteria and differential metabolites in fecal samples. The results showed that on day 56, *g_Acidaminococcus* was significantly positively correlated with thiamine, 4-amino-5-hydroxymethyl-2-methylpyrimidine, 5-hydroxyindole-3-acetic acid, and indole-3-acetic acid; *g_Acholeplasmataceae_EMP-G18* with ornithine, creatinine, indole-3-acetic acid, and 5-hydroxyindole-3-acetic acid; and *g_Butyricimonas* with L-threonine, isopropylmaleic acid, and 5-hydroxyindole-3-acetic acid (*p* < 0.05). As shown in Figure 6A on d 63, *g_Leyella* was significantly positively correlated with homocysteine, isopropylmaleic acid; *g_norank_f_Flavobacteriaceae* with homocysteine, creatine, L-phosphoarginine, spermidine, phosphoserine, 4-(2-amino-3-hydroxyphenyl)-2,4-dioxobutanoic acid, o-succinyl-l-homoserine, 5-hydroxykynurenine, 5-hydroxyindoleacetaldehyde, N-Formylkynurenine, 3-isopropylmalic acid; isopropylmaleic acid; and *g_Butyricicoccaceae-UCG-009* with o-succinyl-l-homoserine, citric acid, homocysteine (Figure 6B *p* < 0.05).

## 4 Discussion

Early research established the importance of vitamin D_3_ for normal calf growth and development (Bechdel et al., 1938). However, as a metabolite of vitamin D₃, 25-hydroxyvitamin D₃ possesses higher bioavailability and has been shown to enhance growth in non-ruminant animals (Zhou et al., 2022). Our results showed that the supplementation of 25D in calves’ diets improved body weight, starter intake, ADG, body diagonal length, and heart girth. These findings align with previous research, McCue et al. (2019) reported that adequate serum 25(OH)D₃ levels enhanced ADG in nursery pigs, likely through supporting bone mineral deposition. Similarly, Xu et al. (2021) observed improved growth performance in calves supplemented with 25(OH)D₃. We note that the improved ADG and body weight in the present study were accompanied by a significant increase in starter intake. This suggests that supplemented 25(OH)D₃ positively influences feed consumption during the critical weaning transition. In conclusion, dietary supplementation of 25(OH)D₃ can effectively promote the growth performance of calves.

Ensuring adequate vitamin D status is particularly crucial for young calves, given their rapid growth and high metabolic demand (Rajaraman et al., 1997; Nonnecke et al., 2009). In the present study, the supplementation of 25(OH)D₃ significantly altered the serum circulating vitamin D metabolites in calves. Specifically, the 25D group exhibited elevated concentrations of both 25(OH)D_3_ and its active metabolite, 1,25(OH)₂D_3_, confirming the effective absorption and enhanced downstream conversion of the supplemented 25(OH)D₃. However, due to alterations in diet and feeding environment during weaning, calves may experience oxidative stress, leading to impairment of intestinal barrier function and reduced in their antioxidant capacity. A previous study reported that dietary 25(OH)D₃ supplementation was associated with enhanced antioxidant capacity and reduced proinflammatory cytokines during weaning (Xu et al., 2021). In this study, dietary 25(OH)D₃ supplementation significantly increased SOD activity and significantly decreased MDA levels on day 63 compared to the control, indicating that 25D supplementation alleviates oxidative stress in weaned calves through enhanced antioxidant capacity. Wang et al. (2022) reported that dietary supplementations of 12,000 IU 25(OH)D_3_ had positive effects on antioxidant capacity, which is consistent with our findings. Meanwhile, in the present study, supplementation of 25(OH) D_3_ in the diet reduced the serum concentrations of IL-1β, IL-6, and TNF-*α* after weaning, which indicates that it alleviates the oxidative stress-induced inflammatory response in weaned calves. Our study found that 25(OH)D₃ supplementation significantly reduced serum D-lactate concentration, likely due to elevated 1,25(OH)₂D₃ levels improving intestinal epithelial integrity. This result is consistent with the report by Gao et al. (2025), in which a combination of sodium butyrate and vitamin D₃ alleviated the increase in D-lactate in cold-stressed broilers. Collectively, the significant increases in body weight, body diagonal length, and heart girth observed in the 25D group of calves after weaning may be attributed to their elevated 25(OH)D₃ levels, further enhancing antioxidant capacity, alleviating inflammation, and reducing intestinal permeability.

In this study, we evaluated the longitudinal changes in the development of fecal microbiota in pre-and postweaning calves. The effect of 25D supplementation on the microbial composition in calves was analyzed using high-throughput 16S rRNA gene sequencing. Additionally, the period from birth to weaning of calves is a critical period for the development and colonization of the intestinal microbiota (Huang P. et al., 2024; Huang Q. et al., 2024). Evidence suggests the beneficial relationship between the intestinal microbiota, their metabolic byproducts, and the maintenance of intestinal homeostasis (Clark and Mach, 2016; Guo et al., 2022). There was no difference in the alpha and beta diversity of fecal microbiota during the pre-postweaning period, indicating no effect of 25(OH)D_3_ supplementation on the diversity of fecal microbiota in calves. However, feces microbiota composition in 25D was clearly different from that in control, which indicated that 25D may play an important role in the regulation of feces microbiota composition of calves. To identify differences in bacterial composition, we utilized the LEfSe analysis method to ascertain differences in feces microbiota in the pre and post weaning. In the control group during the pre-weaning phase, high abundances of *Alistipes* and *Rhodospirillales* were observed, both of which are bacteria characterized by their pro-inflammatory properties. *Alistipes* is a Gram-negative, obligately anaerobic genus of bacteria belonging to the family *Rikenellaceae* within the phylum *Bacteroidota*. Metwaly et al. (2023) reported that introducing segmented filamentous bacteria into specific pathogen-free housed mice induced severe intestinal inflammation and substantially increased the relative abundance of *Alistipes*. Furthermore, Moschen et al. (2016) found that *Alistipes*, enriched in the inflamed colon under Lcn2-deficient conditions, robustly promotes both inflammation and tumor development in mice. *Rhodospirillales* belong to the phylum Proteobacteria. Sbierski-kind et al. (2022) reported that the AdLib microbiota in gnotobiotic mice was enriched with *Rhodospirillales*, which correlated with elevated levels of memory/effector T cells and memory B cells in the colon and spleen, and associated with obesity and related pathologies (Sbierski-Kind et al., 2022). Therefore, the lower abundance of *Alistipes* and *Rhodospirillales* in the 25D group directly reduced their pro-inflammatory potential. *Acidaminococcus* is known to utilize amino acids, particularly glutamic acid, as its exclusive energy substrate to produce acetic and butyric acids, which were negatively correlated with metabolic diseases (Rogosa, 1969; Li et al., 2021; Ponziani et al., 2023). Both *Frisingicoccus* and *Lachnospiraceae_NK4A136*, which belong to the family *Lachnospiraceae*, have been reported in previous studies to be associated with the production of butyric acid and the maintenance of intestinal health (Chen et al., 2023; Feng et al., 2023). In this experiment, we also observed significantly lower D-lactate concentrations in the 25D group on days 56 and 63. Serum D-lactate can reflect intestinal mucosal barrier function (Qing et al., 2019). During the post-weaning phase, the enrichment of butyrate-producing genera, including *Ruminococcus* and *Erysipelotrichaceae_UCG-003*, was observed in the 25D group. *Ruminococcus, which* mainly decomposes complex sugars and cellulose, promotes butyrate acid production (Rajilić-Stojanović and De Vos, 2014). *Erysipelotrichaceae_UCG-003*, a member of the butyrate-producing *Erysipelotrichaceae* family, functions through its role in regulating intestinal immunity (Xiaoyan et al., 2018; Huang P. et al., 2024; Huang Q. et al., 2024). As postulated by Xin et al. (2021), these taxa may serve as probiotic features that positively influence intestinal health in calves. *Leyella* belongs to the family *Prevotellaceae*. Previous studies have reported that this genus utilizes substrates synergistically with *Blautia* and is associated with acetic acid production and intestinal microbial ecosystem balance (Precup and Vodnar, 2019; Wong et al., 2024). Research showed that an increased abundance of *Barnesiellaceae* has been associated with metabolic disorders in other species, its rise in the calf microbiota may also signify a similar dysbiotic state (Tong et al., 2018). Moreover, Arce-cordero et al. (2022) observed a negative correlation of *Christensenellaceae_R-7_group* with propionate suggests that its increased abundance may restrict the availability of this major gluconeogenic precursor, potentially compromising energy metabolism and growth. Therefore, the lower abundance of *Barnesiellaceae*, *Christensenellaceae_R-7_group* suggested that 25D may help calves resist intestinal damage and growth challenges caused by weaning stress by modulating their intestinal microbiota. In summary, 25D optimizes the structure of the intestinal microbiota bacey modulating its composition, selectively inhibiting harmful bacteria and promoting the proliferation of beneficial bacteria, thereby enhancing calf growth and development.

Given that 16S rRNA sequencing revealed significant compositional changes in the gut microbiota, we subsequently conducted fecal metabolomics analysis to explore metabolite changes within the gut microenvironment. Variations in feces metabolite levels are closely associated with host health (Liufu et al., 2024). Metabolomic analysis revealed significant differences in feces metabolites between the 25D and control groups, specifically amino acids and thiamine. The elevated levels of metabolites in feces may result from enhanced host synthesis or increased microbial metabolic activity in the intestine. This indicates an overall upregulation of the body’s metabolism. Tryptophan, and its metabolites, such as 5-hydroxytryptamine (5-HT) and indole, possess regulatory effects on host immunity, intestinal barrier function, and oxidative stress (Lin et al., 2022; Tan et al., 2022). As the terminal metabolites of serotonin and indole, respectively, elevated levels of 5-Hydroxyindole-3-Acetic Acid (5-HIAA) and indole-3-acetic acid indicate increased activity in two distinct tryptophan-derived metabolic pathways. Therefore, 25D supplementation may promote tryptophan metabolism in pre-weaning calves, thereby exerting an effect on maintaining intestinal health. Studies have shown that the large intestine can absorb thiamine through specialized transport systems. These absorbed vitamins not only serve as nutrients for the host but also participate in the regulation of the immune system (Said and Mohammed, 2006; Uebanso et al., 2020). Most amino acids from the mammalian diets are primarily absorbed in the small intestine (Elolimy et al., 2020). Therefore, the observed downregulation of fecal amino acid metabolites may imply improve intestinal absorption, which could be considered a favorable indicator of digestive health. Studies have indicated that inflammation disrupts the protection of the epithelial barrier, leading to impaired absorption of nutrients in the intestinal and consequently elevated levels of amino acids in the feces of IBD patients (Bauset et al., 2021). The results of the present experiment showed that the down-regulation of amino acid metabolites in the feces of the 25D group indicated that the intestinal digestion and absorption efficiency were improved. Spermidine, a polyamine with autophagy inducing activity, confers crucial benefits in patients with aging-related diseases and metabolic dysfunction. Previous studies shown that adding spermidine can downregulation of serum DAO and D-lactate concentrations, enhance intestinal barrier integrity (Ma et al., 2020). Therefore, the lower D-lactate in this study may be related to higher spermidine in the serum.

To determine whether local intestinal metabolic changes further affect systemic metabolism, we additionally performed serum metabolomics. Arachidonic acid (AA) and its metabolites have attracted in relation to inflammatory processes and disease (Sala et al., 2018). In serum, specific phospholipids serve as reservoirs for AA. Notably, both Gpcho (20:1/20:4) and PC (34:1) are classified as lecithin and serve as important precursors for the synthesis of AA. The downregulation of serum lecithin levels is consistent with the decreased content of AA, indicating that the activity of the arachidonic acid metabolic pathway may be suppressed. As a metabolite of Leukotriene B₄, 12(S)-Leukotriene B4 has significantly reduced combining capacity to LTB₄ receptors and decreased pro-inflammatory activity. The upregulation of this metabolite indicates the attenuation of leukotriene-mediated inflammatory responses (Jin et al., 1998). Moreover, the alpha-linoleic acid pathway is the core pathway for the synthesis of unsaturated fatty acids. Studies have shown that upregulation of stearidonic acid is negatively correlated with cardiovascular diseases, inflammation, cancer, and neurological disorders (Whelan, 2009). Thereby, the upregulation of stearidonic acid reflects enhanced biosynthesis of anti-inflammatory polyunsaturated fatty acids (PUFA). The downregulation in alpha-tocopherol and gamma-tocotrienol levels can be attributed to stabilizing the elevated stearidonic acid, as these antioxidants play a critical role in preventing PUFA peroxidation (Opgenorth et al., 2020). Therefore, the higher level of stearidonic acid observed in the 25D group suggests that 25D can effectively mitigate the inflammatory response by enhancing this key pathway. Based on the results of KEGG pathway topology analysis, we found that 25D improved growth performance mainly by modulating amino acid and carbohydrate metabolism pathways during the post-weaning period. As illustrated in the clustering heatmap, citrate, fumarate, and (s)-malate are key intermediates in the TCA cycle. Moreover, the upregulation of D-fructose-1P accelerates the glycolysis pathway, thereby providing more acetyl-CoA for the TCA cycle. Therefore, the increase of citrate, fumarate, and (s)-malate may be the main reason for promoting energy metabolism in the 25D group, thus promoting body weight and ADG. L-proline can be synthesized from L-ornithine through catalysis by aminotransferase and pyrroline-5-carboxylate reductase, and the resulting NADP+ can enter pentose phosphate pathway to regenerate NADPH, thereby supporting cellular redox defense (Liu and Phang, 2012). Additionally, studies have shown a significant downregulation in the ectoine biosynthesis pathway in calves infected with IBRV (Yi et al., 2025). Therefore, the lower inflammatory factors and the higher SOD in this study may be related to higher L-proline and ectoine levels in the serum.

Finally, we used the Mantel test to analyze correlations between microorganisms and metabolites, thereby identifying which specific microbial taxa are functionally associated with key metabolic changes in feces. The correlation analysis revealed that, among the many differentially fecal bacteria, *g_Acidaminococcus* and *g_Leyella* were positively correlated with amino acid metabolites. Specifically, pre-weaning *g_Acidaminococcus* was positively correlated with thiamine, 4-amino-5-hydroxymethyl-2-methylpyrimidine, 5-hydroxyindole-3-acetic acid, and indole-3-acetic acid. Post-weaning *g_Leyella* was positively correlated with homocysteine, isopropylmaleic acid. This suggested that *g_Acidaminococcus* and *g_Leyella* may serve as key microbial biomarkers influencing the fecal amino acid metabolism profile, and that 25D could enhance this metabolic pathway by promoting the proliferation of such beneficial bacteria, thereby ultimately improving intestinal health. Several factors limit the generalizability of our findings, including the use of a single farm, a single season (spring), and only Holstein heifers. Future studies involving multiple farms, different seasons, various breeds, and male calves are needed to validate and extend our results.

## 5 Conclusion

In summary, the current investigation showed that calves’ growth performance, starting intake, antioxidant capacity, and inflammatory response well all greatly improved by dietary 25D supplementation. The primary mediators of these beneficial effects are alterations in the composition of the intestinal microbiota, particularly the enrichment of *Bacillota* and *Bacteroidota*, which in turn boosts the production of microbial metabolites such as SCFA. Metabolomic analysis suggested that feeding 25D diets affected amino acid metabolic pathways and citrate cycle, which contributed to the alteration of metabolites in pathways alanine, aspartate and glutamate metabolism, cysteine and methionine metabolism, tryptophan metabolism, arginine and proline metabolism, and citrate cycle. These metabolites promote intestinal homeostasis and boost antioxidant capacity, thus playing a crucial role in alleviating weaning-induced stress. Overall, 25D effectively alleviated weaning stress and promoted the growth and development of calves by inhibiting inflammation, reducing intestinal permeability, and promoting beneficial bacterial growth.

## Data Availability

The data presented in the study are deposited in the NCBI Sequence Read Archive (SRA) under BioProject, accession number PRJNA1468380.

## Footnotes

1
https://huttenhower.sph.harvard.edu/LEfSe

2
https://www.Genome.jp/kegg/
